# Right-side diaphragmatic eventration with atrial septa defect and cleft palate in an infant: a case report

**DOI:** 10.1186/s13256-023-03910-4

**Published:** 2023-04-21

**Authors:** Mansoor Aslamzai, Fazal Rahman Rahmani, Turyalai Hakimi, Abdul Hakim Mukhlis, Basir Ahmad Froogh

**Affiliations:** 1 Department of Neonatology, Kabul University of Medical Sciences, 3rd District, Kabul, 1003 Afghanistan; 2 Department of Abdominal Surgery, Kabul University of Medical Sciences, 3rd District, Kabul, 1003 Afghanistan; 3 Department of Pediatrics Surgery, Kabul University of Medical Sciences, 3rd District, Kabul, 1003 Afghanistan

**Keywords:** Diaphragmatic eventration, ASD and cleft palate

## Abstract

**Background:**

Congenital right-side diaphragmatic eventration with atrial septal defect and cleft palate is a rare congenital anomaly.

**Case presentation:**

We present a rare case of congenital right-sided diaphragmatic eventration along with atrial septal defect, cleft palate, pneumonia, and undernutrition in a 3-month-old Asian and Afghan girl. The clinical features were observed in the third month of life, and the diagnosis of these anomalies was established by the patient’s history, physical examination, chest X-ray, thoracic computed tomography, and echocardiography. Her condition was good after supportive treatment. Since the index case of diaphragmatic eventration was associated with congenital heart disease, cleft palate, and parental consanguinity, a genetic basis may have played an important role in the pathogenesis of this anomaly.

**Conclusion:**

Eventration of the diaphragm may be diagnosed in early infancy, and genetic factors may contribute to its pathogenesis.

## Background

Diaphragmatic eventration is characterized by an abnormal partial or total elevation of an intact diaphragm due to the abnormality of its musculature. This anomaly can be congenital or acquired, with an estimated prevalence of 1 per 10,000 live births, and usually occurs on the left side [1–3]. Congenital eventration is caused by an underdeveloped diaphragmatic muscle or a lack of the phrenic nerve. The most common cause of acquired eventration is phrenic nerve injury, which can result from either a traumatic birth or thoracic surgery for congenital heart disease [4]. Focal diaphragm elevation is very rare and has generally been observed on the anteromedial right hemidiaphragm, while complete elevation has been found on the left hemidiaphragm [5]. The manifestation of diaphragmatic eventration can be ranged from asymptomatic, respiratory, and gastrointestinal discomforts to a potentially fatal rupture [2, 4–7]. Radiographic imaging such as chest X-ray and computed tomography is used to confirm the diagnosis, and treatment usually consists of supportive care and, in rare situations, surgical management [4].

## Case report

**Fig. 1:**
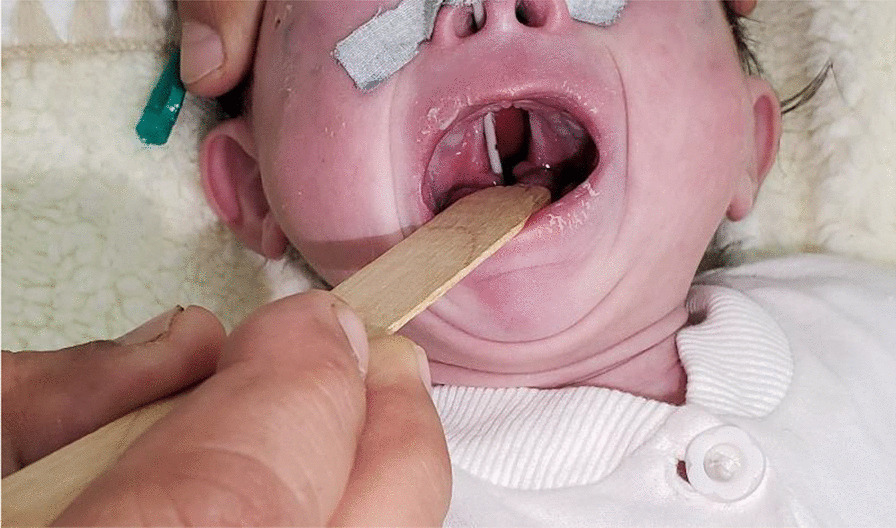
Cleft palate is obviously visible in the index infant

**Fig. 2:**
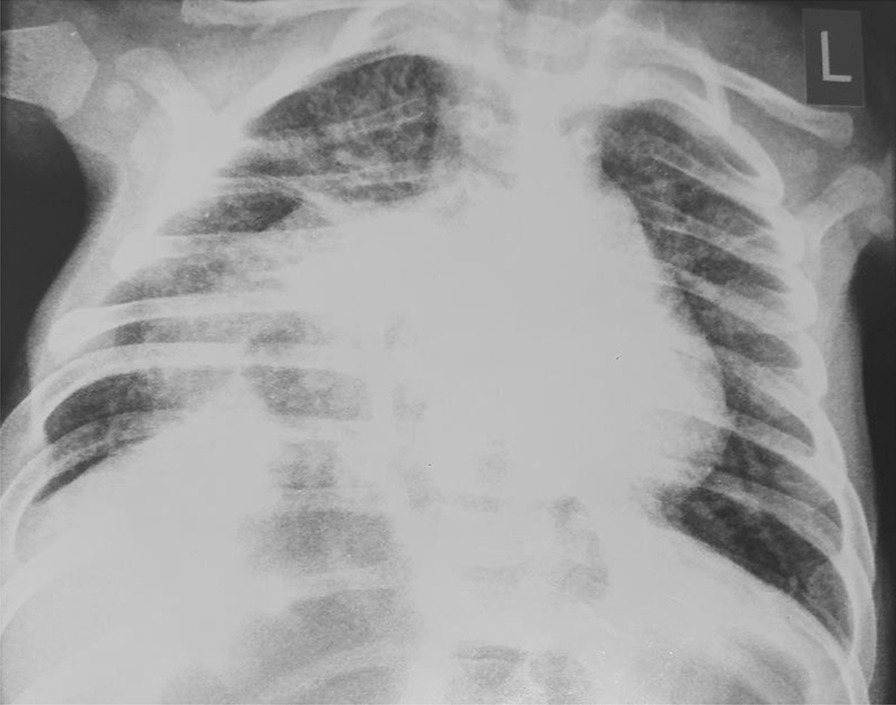
Before the management of bacterial pneumonia, chest radiography shows an elevation of right hemidiaphragm at the medial part and right lung infiltration in the index baby

**Fig. 3:**
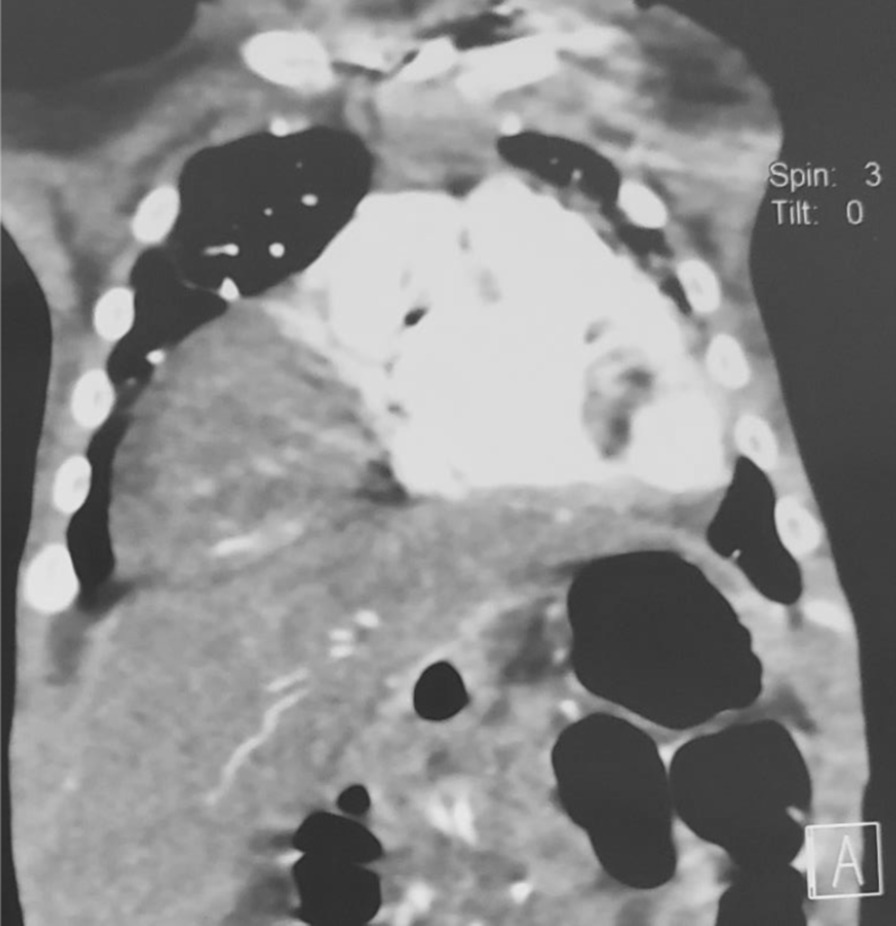
Front view of the thorax on contrast-enhanced computed tomography demonstrates focal elevation at the medial portion of right hemidiaphragm containing abdominal viscera

**Fig. 4:**
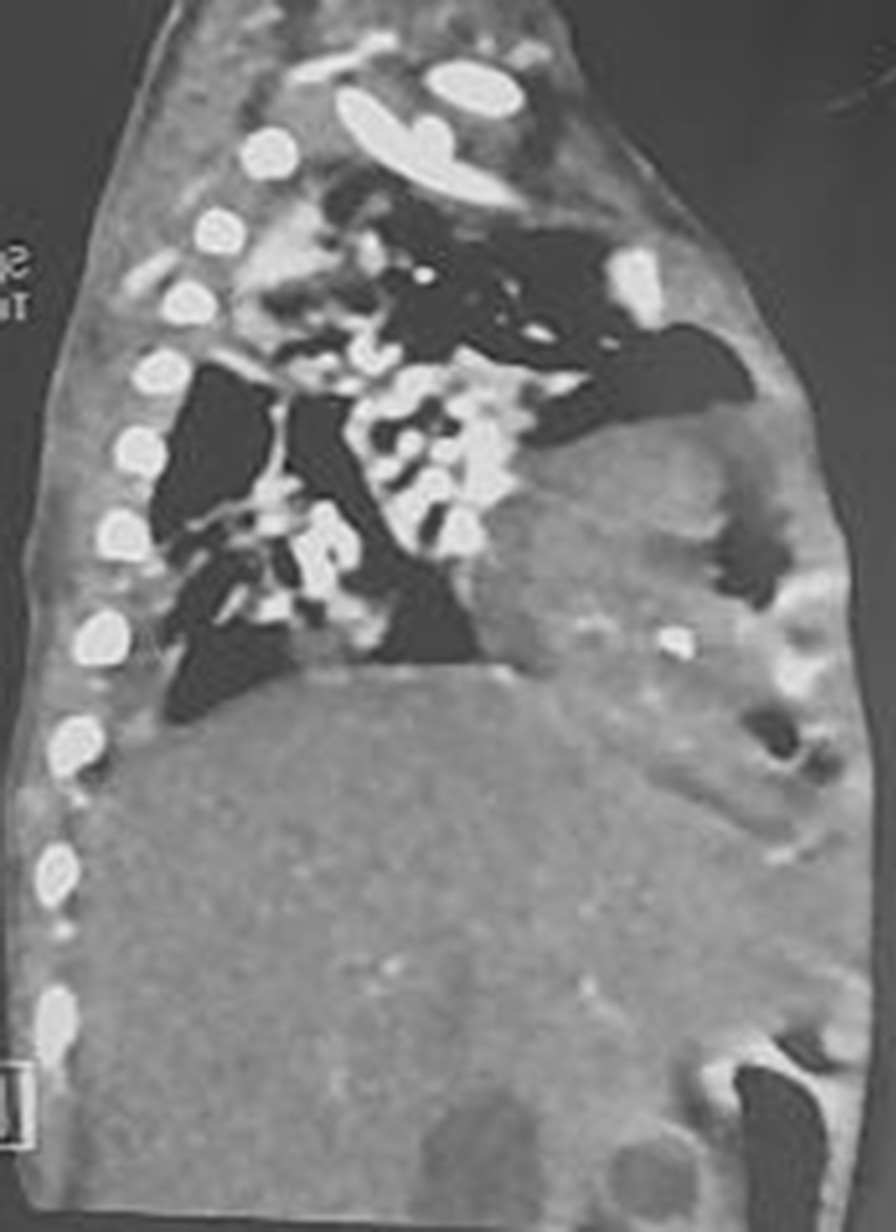
Lateral view of the thorax on contrast-enhanced computed tomography shows elevation at the anterior portion of right hemidiaphragm containing abdominal viscera

**Fig. 5:**
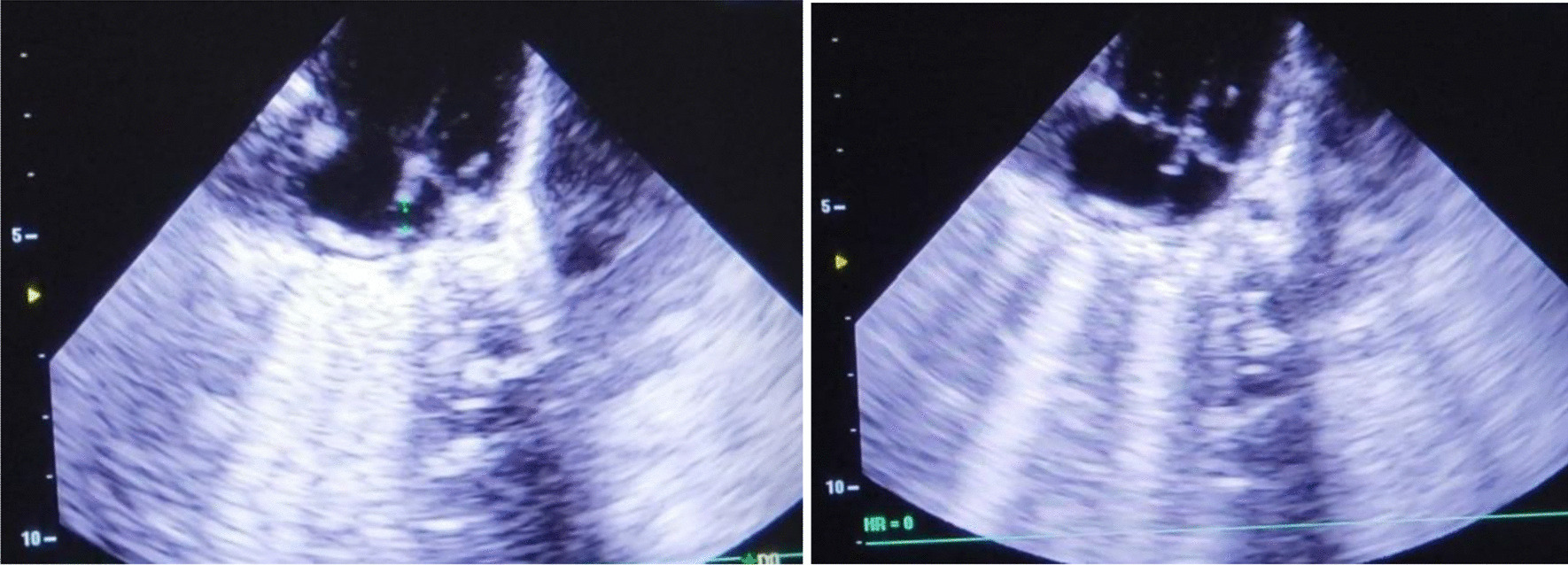
Doppler echocardiography reveals an atrial septal defect in the index infant

**Fig. 6:**
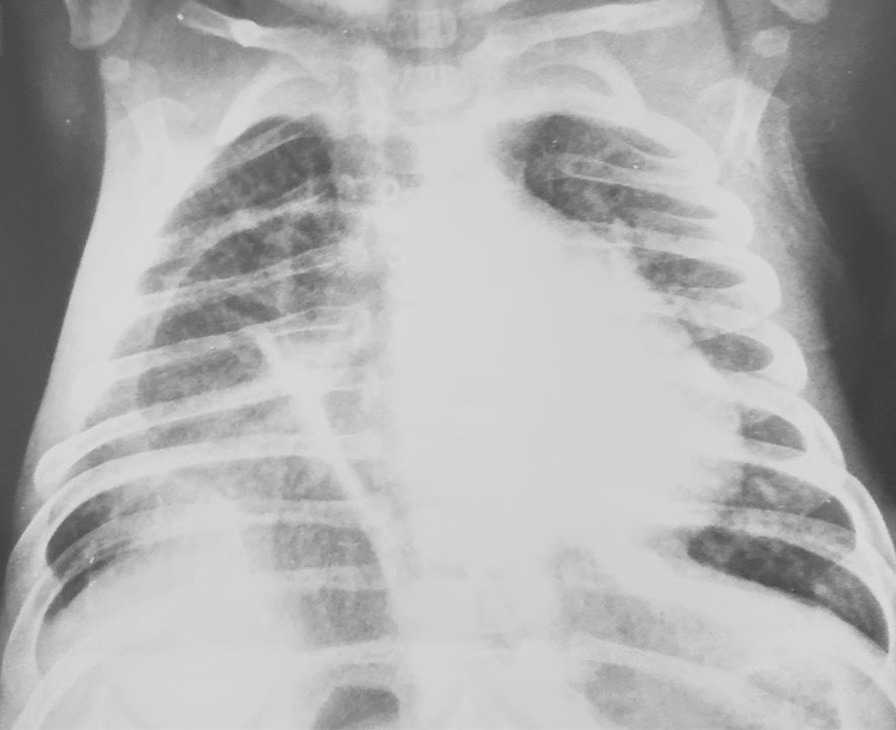
After the treatment of bacterial pneumonia, chest radiography shows elevation of right hemidiaphragm at the medial part and decrease of right lung infiltration in infant

A 3-month-old female infant weighing 3.5 kg was born normally at 38 weeks of gestation to a 39-year-old Asian and Afghan multigravida mother by cesarean delivery at a local hospital. The mother was in good health during pregnancy and did not receive any teratogenic drugs or radiation. The baby had no history of trauma or surgery. The parents were third-degree relatives, which denotes parental consanguinity. The infant was admitted to the intensive care unit (ICU) of the pediatric department due to respiratory difficulty, poor sucking, and fever. On physical examination, lethargy, a length of 55 cm, rectal temperature of 40 °C, respiratory rate of 95/minute, heart rate of 190/minute, subcostal and intercostal retraction, nasal flaring, and central cyanosis with oxygen saturation of 74% were detected. Chest auscultation revealed crackles, diminished breathing sounds, and bowel sound in the lower right thorax. Furthermore, a cleft palate was also found during physical examination (Fig. 1). Blood investigations revealed hemoglobin of 12.8 gm/dl, total leukocyte count of 16,500/mm^3^ (polymorphs 79.9%, lymphocytes 16.1%, eosinophils 0.2%, monocytes 3.3%, and basophil 0.5), platelet count of 559,000/mm^3^, and C-reactive protein of 12 mg/dl. Arterial blood gas analysis showed pH (7.39), pCO_2_ (32 mmHg), pO_2_ (59.4 mmHg), and bicarbonate (20 meq/l). The liver and renal function tests were within normal limits. The chest X-ray demonstrated prominent elevation on the right side of the diaphragm with infiltration in the right lung (Fig. 2). The above mentioned abnormal respiratory findings, fever, leukocytosis, elevated C-reactive protein level, and the radiographic findings were used to diagnose bacterial pneumonia and right-sided diaphragmatic eventration. Based on the weight-for-length reference card, this infant of 3.5 kg weight and 55 cm length classified as moderate acute malnutrition. The patient was put on intravenous fluids, electrolytes, oxygen, and antibiotics, consisting of ceftriaxone intravenously. On the fourth day of admission, the baby became stable with a rectal temperature of 37.5 °C, respiratory rate of 60/minute, heart rate of 140/minute, and no intercostal retraction. Therefore, we started high protein and calorie feeding via a nasogastric tube and advised computed tomography (CT) of the chest for the confirmation of diaphragmatic eventration. The contrast-enhanced CT scan revealed focal elevation at the anteromedial portion of the right hemidiaphragm, including parts of the liver and transverse colon (Figs. 3, 4), all of which are diagnostic of congenital right-side diaphragmatic eventration. On the fifth day of admission, Doppler echocardiography revealed an ostium secundum type of atrial septal defect (ASD) with a 4 mm diameter (Fig. 5) and a pulmonary to systemic blood flow ratio (Qp/Qs) of less than 1.5. Finally, the patient was diagnosed as a case of congenital right-side diaphragmatic eventration, a small ostium secundum type of ASD, cleft palate, bacterial pneumonia, and moderate acute malnutrition. Bacterial pneumonia was treated by intravenous antibiotic for 10 days. The mother’s breast milk and a high protein and calorie formula were used to treat moderate acute malnutrition. A pulmonary to systemic blood flow ratio (Qp/Qs) of less than 1.5 was accompanied by a small-size ASD, hence no surgical management was necessary. Palatoplasty or surgical repair for cleft palate is usually performed at 9–12 months of age. On the ninth day of admission, she was stable with full enteral feeding and no need for respiratory support. The lung infiltration was decreased as shown in the second chest X-ray (Fig. 6). The indications of surgical intervention for diaphragmatic eventration are the presence of respiratory distress despite medical management or respiratory failure. Therefore, such management was not performed for the index case.

## Discussion

Eventration of the diaphragm is a rare malformation that usually develops unilaterally on the left side [1–3]. It was identified for the first time in 1774 by Jean Louis Petit during a postmortem investigation, and the term was first used by Beclard in 1829 [6, 7]. Congenital eventration can be caused by either a lack of diaphragmatic muscle or a phrenic nerve deficit, and replacement of the diaphragmatic muscle with fibroelastic tissue [4]. The indications for surgical intervention for diaphragmatic eventration are the presence of respiratory distress despite medical management or respiratory failure [8, 9]. Persistent interatrial communication is called an atrial septal defect (ASD) and has three types: ostium secundum, ostium primum, and sinus venosus. The pulmonary to systemic blood flow ratio (Qp/Qs) needs to be greater than 2.0 to indicate surgical intervention for ASD [10, 11]. Cleft palate is defined as a congenital abnormal gap in the upper palate. The etiology is complex including genetic factors [12]. Palatoplasty is performed at about 9–12 months of age [10]. Bacterial pneumonia is the infectious inflammation of lung parenchyma leading to cough, fever, tachypnea, chest retraction, leukocytosis, hypoxia, elevated C-reactive protein, and lung infiltration on chest X-ray. Respiratory support and antibiotic therapy are the mainstays of management [14]. Acute malnutrition is defined as low weight-for-height and is classified as moderate and severe types. A high protein and calorie diet is the treatment of choice [15].

According to the evidence of clinical, chest X-ray, and thoracic computed tomography findings, the index case is a congenital right-sided diaphragmatic eventration along with a small-sized ASD, cleft palate, bacterial pneumonia, and moderate acute malnutrition. The cleft palate was clearly visible at the upper palate of the baby (Fig. 1). Diagnostic findings of diaphragmatic eventration on chest radiography and thoracic computed tomography were the focal elevation at the anteromedial section of right hemidiaphragm containing components of liver and transverse colon (Figs. 2, 3, 4). The eventration of diaphragm was identified during early infancy, without a history of trauma or surgical intervention, hence it is a congenital type. A small size of ASD (Fig. 5) with a pulmonary to systemic blood flow ratio (Qp/Qs) of less than 1.5 was diagnosed by Doppler echocardiography. The presence of ASD and cleft palate with diaphragmatic eventration in our case highlights a significant difference from previously reported cases [2, 4–7].

The current case demonstrated triple congenital anomalies and positive parental consanguinity, suggesting a possible role of genetics in the development of diaphragmatic eventration. According to the study Wynn *et al*. multiple genetic variants are attributed to the development of diaphragmatic hernia [16]. Wojcik and Agrawal concluded that congenital abnormalities can result from a range of genetic variations [17]. These findings support our hypothesis regarding the genetic basis in the pathogenesis of diaphragmatic eventration. The clinical features of the infant presented in this case report were respiratory distress, signs of respiratory infection, and slow weight gain, which are consistent with the literature [2, 4–7]. The patient had a stable condition on full enteral feeding without any respiratory support after 8 days of hospitalization. These findings are similar to the study of Groth and Andrade [18].

## Conclusions

The manifestations of diaphragmatic eventration may be observed during the first few months of life. The index case of congenital right-side diaphragmatic eventration was associated with atrial septal defect and cleft palate and had parental consanguinity. Therefore, in the pathogenesis of this anomaly, a genetic basis may be implicated. Further analytic studies are needed to evaluate this issue. Since bacterial pneumonia and undernutrition accompanied the current case, the proper evaluation and management of pulmonary infection and nutritional disorder are suggested for infants with diaphragmatic eventration.

## Data Availability

The documents used during the current study are available from the corresponding author on reasonable request.

## Abbreviations

ASD: Atrial septal defect
CT: Computed tomography
ICU: Intensive care unit

## References

[1] Guliyeva A, Jafarova A, Nazarova I, Hasanova A, Sardarova E (2021). Fetal diaphragmatic eventration. Ultrasound Obstetr Gynecol.

[2] Shah NN, Mohsin M, Khursheed SQ (2008). Eventration of diaphragm with gastric volvulus: a case report. Cases J.

[3] Al-Salem A. Eventration of the diaphragm. In An illustrated guide to pediatric surgery. Springer, Cham; 2014, pp 345–349. 10.1007/978-3-319-06665-3_47.

[4] Souza-Gallardo LM, Centellas-Hinojosa S, Parra-Flores M (2016). Diaphragmatic eventration in adults. Case report. Rev Fac Med UNAM.

[5] Kang H, Lee S, Park H (2019). Ultrasound-guided perioperative management of 28-month-old patient with congenital diaphragmatic eventration. SAGE Open Med Case Rep..

[6] Carrasco A, Castro R (2018). Right diaphragmatic eventration with an intrathoracic kidney: case report and review of the literature. Case Rep Surg.

[7] Lone R, Sharma M, Wani M (2009). Traumatic diaphragmatic rupture, a diagnostic dilemma in the presence of eventration: a case report. Cases J.

[8] Sallout B, Alshebli D, Sallout L, Al Baqawi B, Faden MS (2021). Fetal diaphragmatic eventration: a case report. J Obstet Gynaecol Can.

[9] Ozken S, Yazici U, Aydin E, Karaoğlanoğlu N (2016). Is surgical plication necessary in diaphragm eventration?. Asian J Surg.

[10] Martin SS, Shapiro EP, Mukherjee M (2015). Atrial septal defects—clinical manifestations, echo assessment, and intervention. Clin Med Insights Cardiol..

[11] Saito T, Ohta K, Nakayama Y, Hashida YH, Maeda A, Maruhashi K, Yachie A (2012). Natural history of medium-sized atrial septal defect in pediatric cases. J Cardiol.

[12] Tarun V, Prabhakar G, Sachin K, Rajat G, Tanu G, Preet HS (2020). Cleft of lip and palate: a review. J Fam Med Primary Care.

[13] Ruiz-Guillén A, Suso-Ribera C, Romero-Maroto M (2021). Perception of quality of life by children and adolescents with cleft lip/palate after orthodontic and surgical treatment: gender and age analysis. Prog Orthod..

[14] Crame E, Shields MD, McCrossan P (2021). Paediatric pneumonia: a guide to diagnosis, investigation and treatment. Paediatr Child Health.

[15] Dipasquale V, Cucinotta U, Romano C (2020). Acute malnutrition in children: pathophysiology, clinical effects and treatment. Nutrients.

[16] Wynn J, Yu L, Chung WK (2014). Genetic causes of congenital diaphragmatic hernia. Semin Fetal Neonatal Med.

[17] Wojcik MH, Agrawal PB (2020). Deciphering congenital anomalies for the next generation. Cold Spring Harb Mol Case Stud..

[18] Groth SS, Andrade RS (2009). Diaphragmatic eventration. Thorac Surg Clinic.

